# A smoothing inexact Newton method for variational inequalities with nonlinear constraints

**DOI:** 10.1186/s13660-017-1433-9

**Published:** 2017-07-10

**Authors:** Zhili Ge, Qin Ni, Xin Zhang

**Affiliations:** 1 College of Science, Nanjing University of Aeronautics and Astronautics, Nanjing, 211106 China; 2 Basic Sciences Department, Nanjing Polytechnic Institute, Nanjing, 210048 China; 3 School of Arts and Science, Suqian College, Suqian, 223800 China

**Keywords:** variational inequalities, nonlinear constraints, inexact Newton method, global convergence, local quadratic convergence

## Abstract

In this paper, we propose a smoothing inexact Newton method for solving variational inequalities with nonlinear constraints. Based on the smoothed Fischer-Burmeister function, the variational inequality problem is reformulated as a system of parameterized smooth equations. The corresponding linear system of each iteration is solved approximately. Under some mild conditions, we establish the global and local quadratic convergence. Some numerical results show that the method is effective.

## Introduction

We consider the variational inequality problem (VI for abbreviation), which is to find a vector $x^{*}\in\Omega$ such that

1 $$ \text{VI}(\Omega,F)\quad \bigl(x-x^{*} \bigr)^{\top}F \bigl(x^{*} \bigr)\geq0, \quad\forall x\in\Omega, $$

where Ω is a nonempty, closed and convex subset of ${\mathcal {R}}^{n}$ and *F* is a continuous differentiable mapping from ${\mathcal {R}}^{n}$ into ${\mathcal {R}}^{n}$. In this paper, without loss of generality, we assume that

2 $$ \Omega:= \bigl\{ x\in R^{n}|g(x)\geq0 \bigr\} , $$

where $g:R^{n} \to R^{m}$ and $g_{i}:R^{n} \to R$, $(i\in I=\{1,2,\ldots ,m\})$ are twice continuously differentiable concave functions. When $\Omega =R^{n}_{+}$, VI reduces to the nonlinear complementarity problem (NCP for abbreviation)

3 $$ x^{*}\in R^{n}_{+},\qquad F \bigl(x^{*} \bigr)\in R^{n}_{+},\qquad {x^{*}}^{\top}F \bigl(x^{*} \bigr)=0. $$

Variational inequalities have important applications in mathematical programming, economics, signal processing, transportation and structural analysis [1–3]. So, there are various numerical methods which have been studied by many researchers; e.g., see [4].

A popular way to solve the $\mbox{VI}(\Omega,F)$ is to reformulate (1) to a nonsmooth equation via a KKT system of variational inequalities and an NCP-function. It is well known that the KKT system of $\mbox{VI}(\Omega,F)$ can be given as follows:

4 $$ \textstyle\begin{cases}F(x)-\nabla g(x)^{\top}\lambda=0,\\ g(x)-z=0,\\ \lambda\geq0, z\geq0, \quad \lambda^{\top}z=0, \end{cases} $$

and the NCP-function $\phi(a,b)$ is defined by the following condition:

5 $$ \phi(a,b)=0 \quad\iff \quad a\geq0, b\geq0, ab=0. $$

Then problem (1) and (2) is equivalent to the following nonsmoothing equation:

6 $$ \begin{pmatrix}F(x)-\nabla g(x)^{\top}\lambda\\ g(x)-z\\ \phi(\lambda,z) \end{pmatrix}=0. $$

Hence, problem (1) and (2) can be translated into (6).

We all know that the smoothing method is a fundamental approach to solve the nonsmooth equation (6). Recently, there has been strong interest in smoothing Newton methods for solving NCP [5–12]. The idea of this method is to construct a smooth function to approximate $\phi(\lambda,z)$. In the past few years, there have been many different smoothing functions which were employed to smooth equation (6). Here, we define

7 $$ H(\mu,x,\lambda,z):= \begin{pmatrix}\mu\\ F(x)-\nabla g(x)^{\top}\lambda\\ g(x)-z\\ \Phi(\mu,\lambda,z) \end{pmatrix}, $$

where

8 $$ \Phi(\mu,\lambda,z):= \begin{pmatrix} \varphi(\mu,\lambda_{1},z_{1})\\ \vdots\\ \varphi(\mu,\lambda_{m},z_{m}) \end{pmatrix} $$

and

9 $$ \varphi(\mu,a,b)=a+b-\sqrt{a^{2}+b^{2}+ \mu^{2}}, \quad\forall(\mu,a,b)\in R^{3}. $$

It follows from equations (4)-(9) that $H(\mu ,x,\lambda,z)=0$ is equivalent to $\mu=0$ and $(x,\lambda,z)$ is the solution of (6). Thus, we may solve the system of smoothing equation $H(\mu,x,\lambda,z)=0$ and reduce *μ* to zero gradually while iteratively solving the equation.

Based on the above symmetric perturbed Fischer-Burmeister function (9), Chen et al. [13] proposed the first globally and superlinearly convergent smoothing Newton method. They dealt with general box constrained variational inequalities. And Rui et al. [14] proposed an inexact Newton-GMRES method for a large-scale variational inequality problem under the assumption of linear inequality constraints.

In reality, variational inequalities with nonlinear constraints are more attractive. These problems have wide applications in economic networks [15], image restoration [3, 16] and so on. So, in this paper, under the framework of smoothing Newton method, we propose a new inexact Newton method for solving $\mbox{VI}(\Omega,F)$ with nonlinear constraints, which extends the scope of constraints. We also prove the global and local quadratic convergence and present some numerical results which show the efficiency of the proposed method.

Throughout this paper, we always assume that the solution set of problem (1) and (2), denoted by $\Omega^{*}$, is nonempty. ${\mathcal {R}}_{+}$ and ${\mathcal {R}}_{++}$ mean the nonnegative and positive real sets. Symbol $\Vert \cdot \Vert $ stands for the 2-norm.

The rest of this paper is organized as follows. In Section 2, we summarize some useful properties and definitions. In Section 3, we describe the inexact Newton method formally and then prove its local quadratic convergence. We also give global convergence in Section 4. In Section 5, we report our numerical results. Finally, we give some conclusions in Section 6.

## Preliminaries

In this section, we denote some basic definitions and properties that will be used in the subsequent sections.

### Definition 2.1

The operator *F* is monotone if, for any $u,v \in{\mathcal {R}}^{n}$,

$$(u-v)^{\top}\bigl(F(u)-F(v) \bigr)\geq0; $$

*F* is strongly monotone with modulus $\mu> 0$ if, for any $u,v \in{\mathcal {R}}^{n}$,

$$(u-v)^{\top}\bigl(F(u)-F(v) \bigr)\geq\mu \Vert u-v \Vert ^{2}; $$

*F* is Lipschitz continuous with a positive constant $L>0$ if, for any $u,v \in{\mathcal {R}}^{n}$,

$$\bigl\Vert F(u)-F(v) \bigr\Vert \leq L \Vert u-v \Vert . $$

The following lemma gives some properties of *H* and its corresponding Jacobian.

### Lemma 2.1

*Let* $H(\mu,x,\lambda,z)$ *be defined by* (7). *Assume that* *F* *is continuously differentiable and strongly monotone*, *g* *is twice continuously differentiable concave*, $(\mu^{*},x^{*},\lambda^{*},z^{*})$ *in* ${\mathcal {R}}_{+}\times{\mathcal {R}}^{n}\times{{\mathcal {R}}^{m}}\times{\mathcal {R}}^{m}$ *is the solution of* $H(\mu,x,\lambda,z)=0$, *the rows of* $\nabla g(x^{*})$ *are linearly independent and* $(\lambda^{*},z^{*})$ *satisfies the strict complementarity condition*. *Then*

(i) $H(\mu,x,\lambda,z)$ *is continuously differentiable on* ${\mathcal {R}}_{+}\times{\mathcal {R}}^{n}\times{\mathcal {R}}^{m}\times{\mathcal {R}}^{m}$.
(ii) $\nabla H(\mu^{*},x^{*},\lambda^{*},z^{*})$ *is nonsingular*, *where*
  10 $$\begin{aligned} &\nabla{ H}(\mu,x,\lambda,z)= \begin{pmatrix} 1&0&0&0\\ 0 &\nabla F(x)-\nabla^{2}g(x)^{\top}\lambda& -\nabla g(x)^{\top}& 0\\ 0 &\nabla g(x)& 0 &-I\\ D_{\mu}& 0 &D_{\lambda}& D_{z} \end{pmatrix}, \\ & D_{\mu}=\operatorname{vec} \biggl(-\frac{\mu}{ \sqrt{\lambda_{i}^{2}+z_{i}^{2}+\mu^{2}}} \biggr), \\ & D_{\lambda}=\operatorname{diag} \biggl(1-\frac{\lambda_{i}}{ \sqrt{\lambda_{i}^{2}+z_{i}^{2}+\mu^{2}}} \biggr)\quad (i=1, \ldots, m), \\ & D_{z}=\operatorname{diag} \biggl(1-\frac{z_{i}}{ \sqrt{\lambda_{i}^{2}+z_{i}^{2}+\mu^{2}}} \biggr)\quad (i=1, \ldots, m) \quad \textit{and} \\ & \nabla^{2} g(x)^{\top}\lambda= \sum^{m}_{j=1}\nabla^{2} g_{j}(x)^{\top}\lambda_{j}. \end{aligned}$$

### Proof

It is not hard to show that *H* is continuously differentiable on ${\mathcal {R}}_{++}\times{\mathcal {R}}^{n}\times{\mathcal {R}}^{m}\times{\mathcal {R}}^{m}$. From $H(\mu^{*},x^{*},\lambda^{*},z^{*})=0$, by (7) we get that $\mu^{*}=0$ easily. Since $(\lambda^{*},z^{*})$ satisfies the strict complementarity condition, i.e., $\lambda_{i}^{*}$, $z_{i}^{*}$ are not equal to 0 at the same time, we have that *H* is also continuously differentiable on $(\mu^{*},x^{*},\lambda^{*},z^{*})$. That is, (i) holds.

Now, we prove (ii). Let $q=(q_{1}, q_{2}, q_{3}, q_{4})^{\top}\in{\mathcal {R}}_{+}\times {\mathcal {R}}^{n}\times{\mathcal {R}}^{m}\times{\mathcal {R}}^{m}$, $q_{1}=(q_{11})$, $q_{2}=(q_{21}, q_{22}, \ldots, q_{2n})$, $q_{3}=(q_{31}, q_{32}, \ldots, q_{3m})$, $q_{4}=(q_{41}, q_{42}, \ldots, q_{4m})$, and

11 $$\begin{aligned} \nabla H \bigl(\mu^{*},x^{*},\lambda^{*},z^{*} \bigr) \begin{pmatrix}q_{1}\\q_{2}\\q_{3}\\q_{4} \end{pmatrix}=0. \end{aligned}$$

Hence, we have 
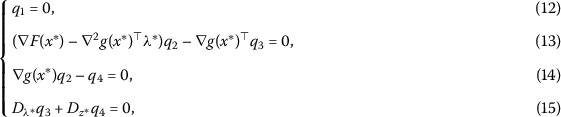
 where

$$ D_{\lambda^{*}}=\operatorname{diag} \biggl(1-\frac{\lambda_{i}^{*}}{ \sqrt{\lambda_{i}^{*2}+z_{i}^{*2}}} \biggr),\qquad D_{z^{*}}=\operatorname{diag} \biggl(1-\frac{z_{i}^{*}}{ \sqrt{\lambda_{i}^{*2}+z_{i}^{*2}}} \biggr). $$

We can observe $q_{1}=0$ easily by (12).

Next, we discuss formula (15). The full form of (15) can be described as follows:

16 $$\begin{aligned} \begin{pmatrix} (1-\frac{\lambda_{1}^{*}}{\sqrt{\lambda_{1}^{*2}+z_{1}^{*2}}} )q_{31}+ (1-\frac{z_{1}^{*}}{\sqrt{\lambda_{1}^{*2}+z_{1}^{*2}}} )q_{41}\\ (1-\frac{\lambda_{2}^{*}}{\sqrt{\lambda_{2}^{*2}+z_{2}^{*2}}} )q_{32}+ (1-\frac{z_{2}^{*}}{\sqrt{\lambda_{2}^{*2}+z_{2}^{*2}}} )q_{42}\\ \vdots\\ (1-\frac{\lambda_{m}^{*}}{\sqrt{\lambda_{m}^{*2}+z_{m}^{*2}}} )q_{3m}+ (1-\frac{z_{m}^{*}}{\sqrt{\lambda_{m}^{*2}+z_{m}^{*2}}} )q_{4m} \end{pmatrix}=0. \end{aligned}$$

According to the strict complementarity condition of $(\lambda^{*},z^{*})$, we have $\lambda_{i}^{*}\geq0$, $z_{i}^{*}\geq0$, $\lambda _{i}^{*}\top z_{i}^{*} =0$ and $\lambda_{i}^{*}$, $z_{i}^{*}$ are not equal to 0 at the same time. If $z_{i}^{*}= 0$, then $\lambda_{i}^{*}> 0$, and

$$ \biggl(1-\frac{\lambda_{i}^{*}}{ \sqrt{\lambda_{i}^{*2}+z_{i}^{*2}}} \biggr)=0,\qquad \biggl(1-\frac{z_{i}^{*}}{ \sqrt{\lambda_{i}^{*2}+z_{i}^{*2}}} \biggr)=1. $$

From (16) we get that $q_{4i}=0$ and $q_{3i}^{\top}q_{4i}=0$. Similarly, if $\lambda_{i}^{*}= 0$, then $z_{i}^{*}> 0$. We get that $q_{3i}=0$ and $q_{3i}^{\top}q_{4i}=0$. Hence, $q_{3}^{\top}q_{4}=0$.

Multiplying the equation of (14) by $q_{3}^{\top}$ on the left-hand side and using $q_{3}^{\top}q_{4}=0$, we have

17 $$ q_{3}^{\top}\nabla g \bigl(x^{*} \bigr) q_{2}=0. $$

Multiplying the equation of (13) by $q_{2}^{\top}$ on the left-hand side and using (17), we have

18 $$ q_{2}^{\top}\bigl(\nabla F \bigl(x^{*} \bigr)- \nabla^{2} g \bigl(x^{*} \bigr)^{\top}\lambda^{*} \bigr)q_{2}=0. $$

Meanwhile, because *F* is strongly monotone, we have that $\nabla F(x^{*})$ is a positive definite matrix. Besides, since *g* is concave and $\lambda^{*}$ is nonnegative, we have that $\nabla^{2} g(x^{*})^{\top}\lambda^{*} = \sum^{m}_{j=1}\nabla^{2} g_{j}(x^{*})^{\top}\lambda^{*}_{j} $ are nonpositive definite matrices, which implies that $(\nabla F(x^{*})- \nabla^{2} g(x^{*})^{\top}\lambda^{*} )$ is a positive definite matrix. So, we get that $q_{2}=0$ by (18).

Substituting $q_{2}=0$ into (13) and using the rows of $\nabla g(x^{*})$ are linearly independent, we get $q_{3}=0$. Substituting $q_{2}=0$ into (14), we get $q_{4}=0$. Hence we have $q=0$, which implies that $\nabla H(\mu^{*},x^{*},\lambda^{*},z^{*})$ is nonsingular. This completes the proof. □

## The inexact algorithm and its convergence

We are now in the position to describe our smoothing inexact Newton method formally by using the smoothed Fischer-Burmeister function (9) to solve the variational inequalities with nonlinear constraints. We also show that this method has local quadratic convergence.

### Algorithm 3.1

Inexact Newton method

Step 0.: *Let* $w=(x,\lambda,z)$ *and* $(\mu^{0},w^{0})\in{\mathcal {R}}_{++}\times{\mathcal {R}}^{n+2m}$ *be an arbitrary point*. *Choose* $\mu^{0}>0$ *and* $\gamma\in(0,1)$ *such that* $\gamma\mu^{0}<\frac{1}{2}$.
Step 1.: *If* $\Vert H(\mu^{k},w^{k}) \Vert ^{2}=0$, *then stop*.
Step 2.: *Compute* $(\Delta\mu^{k},\Delta w^{k})$ *by*
  19 $$ \textstyle\begin{array}{c}H(\mu^{k},w^{k}) \end{array}\displaystyle + \nabla{H} \bigl( \mu^{k},w^{k} \bigr) \begin{pmatrix}\Delta\mu^{k}\\ \Delta w^{k} \end{pmatrix} = \begin{pmatrix}\rho_{k}\mu^{0}\\ r^{k} \end{pmatrix}, $$
  *where* $\rho_{k}=\rho(\mu^{k},w^{k}):=\gamma\min\{1, \Vert H(\mu^{k},w^{k}) \Vert ^{2}\}$, *and* $r^{k} \in{\mathcal {R}}^{n+2m}$ *such that* $\Vert r^{k} \Vert \leq\rho_{k}\mu^{0}$.
Step 3.: *Set* $\mu^{k+1}=\mu^{k}+\Delta\mu^{k}$ *and* $w^{k+1}=w^{k}+\Delta w^{k}$. *Set* $k:=k+1$ *and go to Step* 1.

### Remark 1

1. In theory, we use $\Vert H(\mu^{k},w^{k}) \Vert ^{2}=0$ as a termination of Algorithm 3.1. In practice, we use $\Vert H(\mu^{k},w^{k}) \Vert ^{2}\leq\varepsilon$ as a termination rule, where *ε* is a pre-set tolerance error.
2. It is obvious that we have $\rho_{k}\leq\gamma \Vert H(\mu^{k},w^{k}) \Vert ^{2}$.
3. From (7) and (19), we have $\mu^{k+1}=\rho_{k}\mu^{0}>0$ for any $k\geq0$.

Now, we are ready to analyze the convergence. The quadratic convergence of Algorithm 3.1 is given below.

### Theorem 3.1

*Assume that* $(\mu^{*},w^{*})$ *satisfies* $H(\mu^{*},w^{*})=0$. *Suppose that* $H(\mu,w)$ *satisfies the condition of Lemma* 2.1 *and* $\nabla H(\mu,w)$ *is Lipschitz continuous with the constant* *L*. *Then we have the following conclusions*:

1. *There exists a set* $D\subset{\mathcal {R}}_{+}\times{\mathcal {R}}^{n+2m}$ *which contains* $(\mu^{*},w^{*})$ *such that for any* $(\mu ^{0},w^{0})\in D$, *the iterate points* $(\mu^{k},w^{k})$ *generated by Algorithm* 3.1 *are well defined*, *remain in* *D* *and converge to* $(\mu^{*},w^{*})$;
2. 20 $$ \bigl\Vert \bigl(\mu^{k+1}-\mu^{*},w^{k+1}-w^{*} \bigr) \bigr\Vert \leq \beta \bigl\Vert \bigl(\mu^{k}-\mu ^{*},w^{k}-w^{*} \bigr) \bigr\Vert ^{2}, $$
  *where* $\beta:= (L+16\gamma\mu^{0} \Vert \nabla H(\mu^{*},w^{*}) \Vert ^{2}) \Vert \nabla H(\mu^{*},w^{*})^{-1} \Vert $.

### Proof

According to Theorem 5.2.1 in [17] and Lemma 2.1, we give the proof in detail.

Denote

$$ u= \begin{pmatrix}\mu\\w \end{pmatrix} , \qquad u^{*}=\begin{pmatrix}\mu^{*}\\w^{*} \end{pmatrix},\qquad \Delta u= \begin{pmatrix}\Delta\mu\\ \Delta w \end{pmatrix},\qquad v^{k}=\begin{pmatrix}\rho_{k}\mu^{0}\\r^{k} \end{pmatrix}. $$

By Step 2 of Algorithm 3.1, we have

21 $$ \bigl\Vert v^{k} \bigr\Vert \leq\rho_{k} \mu^{0} + \bigl\Vert r^{k} \bigr\Vert \leq2 \rho_{k}\mu ^{0}\leq2\gamma \mu^{0} \bigl\Vert H \bigl(u^{k} \bigr) \bigr\Vert ^{2}. $$

According to Lemma 2.1, we get that $\nabla H(u^{*})$ is nonsingular. Then there exist a positive constant $\bar{t}<\frac{1}{\beta}$ and a neighborhood $N(u^{*},\bar{t})$ of $u^{*}$ such that $L\bar{t} \leq \Vert \nabla H(u^{*}) \Vert $, and for any $u\in N(u^{*},\bar{t})$, we have that $\nabla H(u)$ is nonsingular and

22 $$\begin{aligned} \bigl\Vert \nabla H(u) \bigr\Vert - \bigl\Vert \nabla H \bigl(u^{*} \bigr) \bigr\Vert \leq \bigl\Vert \nabla H(u)- \nabla H \bigl(u^{*} \bigr) \bigr\Vert \leq L \bigl\Vert u-u^{*} \bigr\Vert , \end{aligned}$$

where the first inequality follows from the triangle inequality and the second inequality follows from the Lipschitz continuity. Hence we have

23 $$ \bigl\Vert \nabla H(u) \bigr\Vert \leq2 \bigl\Vert \nabla H \bigl(u^{*} \bigr) \bigr\Vert $$

for any $u\in N(u^{*},\bar{t})$. Similarly, by the perturbation relation (3.1.20) in [17], we know that $\nabla H(u)$ is nonsingular and

24 $$ \bigl\Vert \nabla H(u)^{-1} \bigr\Vert \leq \frac{ \Vert \nabla H(u^{*})^{-1} \Vert }{1- \Vert \nabla H(u^{*})^{-1} (\nabla H(u)-\nabla H(u^{*}) ) \Vert }\leq2 \bigl\Vert \nabla H \bigl(u^{*} \bigr)^{-1} \bigr\Vert . $$

Besides, for any $t \in[0,1]$, we have

$u^{*}+t(u-u^{*})\in N(u^{*},\bar{t})$ and $H(u)-H(u^{*})=\int_{0}^{1}\nabla H[u^{*}+t(u-u^{*})](u-u^{*})\,dt$.

From $\Vert H(u^{*}) \Vert =0$, we have

25 $$\begin{aligned} \bigl\Vert H(u) \bigr\Vert \leq \int_{0}^{1} \bigl\Vert \nabla H \bigl(u^{*}+t \bigl(u-u^{*} \bigr) \bigr) \bigr\Vert \bigl\Vert \bigl(u-u^{*} \bigr) \bigr\Vert \,dt \leq2 \bigl\Vert \nabla H \bigl(u^{*} \bigr) \bigr\Vert \bigl\Vert u-u^{*} \bigr\Vert . \end{aligned}$$

According to Algorithm 3.1, for any $u^{k} \in N(u^{*},\bar{t}), k\geq0$, we have

26 $$\begin{aligned} & u^{k+1}-u^{*} \\ &\quad = u^{k}-u^{*}-\nabla H \bigl(u^{k} \bigr)^{-1} \bigl(H \bigl(u^{k} \bigr)-v^{k} \bigr) \\ &\quad = \nabla H \bigl(u^{k} \bigr)^{-1} \bigl(\nabla H \bigl(u^{k} \bigr) \bigl(u^{k}-u^{*} \bigr)- \bigl(H \bigl(u^{k} \bigr)-H \bigl(u^{*} \bigr) \bigr)+v^{k} \bigr) \\ &\quad = \nabla H \bigl(u^{k} \bigr)^{-1} \\ &\qquad{}\times \biggl( \int_{0}^{1} \bigl(\nabla H \bigl(u^{k} \bigr)-\nabla H \bigl(u^{*}+t \bigl(u^{k}-u^{*} \bigr) \bigr) \bigr) \bigl(u^{k}-u^{*} \bigr)\,dt+v^{k} \biggr). \end{aligned}$$

Taking norm of both sides, we get

27 $$\begin{aligned} & \bigl\Vert u^{k+1}-u^{*} \bigr\Vert \\ &\quad\leq \bigl\Vert \nabla H \bigl(u^{k} \bigr)^{-1} \bigr\Vert \biggl( \int_{0}^{1}L(1-t) \bigl\Vert u^{k}-u^{*} \bigr\Vert ^{2}\,dt+ \bigl\Vert v^{k} \bigr\Vert \biggr) \\ &\quad\leq 2 \bigl\Vert \nabla H \bigl(u^{*} \bigr)^{-1} \bigr\Vert \biggl(\frac{1}{2}L \bigl\Vert u^{k}-u^{*} \bigr\Vert ^{2} + 2\gamma\mu ^{0} \bigl\Vert H \bigl(u^{k} \bigr) \bigr\Vert ^{2} \biggr) \\ &\quad\leq 2 \bigl\Vert \nabla H \bigl(u^{*} \bigr)^{-1} \bigr\Vert \biggl(\frac{1}{2}L \bigl\Vert u^{k}-u^{*} \bigr\Vert ^{2}+8\gamma\mu ^{0} \bigl\Vert \nabla H \bigl(u^{*} \bigr) \bigr\Vert ^{2} \bigl\Vert u^{k}-u^{*} \bigr\Vert ^{2} \biggr) \\ &\quad = \bigl(L + 16\gamma\mu^{0} \bigl\Vert \nabla H \bigl(u^{*} \bigr) \bigr\Vert ^{2} \bigr) \bigl\Vert \nabla H \bigl(u^{*} \bigr)^{-1} \bigr\Vert \bigl\Vert u^{k}-u^{*} \bigr\Vert ^{2}, \end{aligned}$$

where the first inequality follows from the Lipschitz continuity, the second inequality follows from (21), and the third inequality follows from (25).

According to the definition of *β* and the condition of $\bar{t}<\frac{1}{\beta}$, we get that $u^{k}$ converges to $u^{*}$. Besides, (20) also holds. This completes the proof. □

## The global inexact algorithm and its convergence

Now, we start our globally convergent method by using the global technique in Algorithm 3.1. We choose a merit function $h(\mu,w)=\frac{1}{2} \Vert H(\mu,w) \Vert ^{2}$ and modify $(\Delta\mu^{k}, \Delta w^{k})$ such that

28 $$ -{ \bigl(\Delta\mu^{k}, \Delta w^{k} \bigr)}^{\top}\nabla h \bigl(\mu^{k},w^{k} \bigr)\geq \delta \bigl\Vert \bigl(\Delta\mu^{k}, \Delta w^{k} \bigr) \bigr\Vert \bigl\Vert \nabla h \bigl(\mu^{k},w^{k} \bigr) \bigr\Vert . $$

We use line search to find a step-length $t^{k}\in(0,1]$ such that

29 $$\begin{aligned} & h \bigl(\mu^{k}+t^{k}\Delta \mu^{k},w^{k}+t^{k}\Delta w^{k} \bigr)\leq h \bigl(\mu^{k},w^{k} \bigr)+\bar {\rho}t^{k}\nabla h \bigl(\mu^{k},w^{k} \bigr)^{\top}\bigl(\Delta \mu^{k}, \Delta w^{k} \bigr), \end{aligned}$$

30 $$\begin{aligned} &\nabla h \bigl(\mu^{k}+t^{k}\Delta \mu^{k},w^{k}+t^{k}\Delta w^{k} \bigr)^{\top}\bigl(\Delta\mu ^{k}, \Delta w^{k} \bigr) \geq\bar{\sigma}\nabla h \bigl(\mu^{k},w^{k} \bigr)^{\top}\bigl(\Delta\mu ^{k}, \Delta w^{k} \bigr) \end{aligned}$$

and

31 $$ \mu^{k}+t^{k}\Delta\mu^{k}\in{ \mathcal {R}}_{++},\qquad w^{k}+t^{k}\Delta w^{k} \in {\mathcal {R}}^{n+2m}, $$

where $\bar{\rho}\in(0,0.5), \bar{\sigma} \in(\bar{\rho},1), \delta\in(0,1)$.

### Algorithm 4.1

Global inexact Newton method

Step 0.: *Choose* $(\mu^{0},w^{0})\in{\mathcal {R}}_{++}\times {\mathcal {R}}^{n+2m}$ *to be an arbitrary point*. *Choose* $\gamma\in(0,1)$ *such that* $\gamma \mu^{0}<\frac{1}{2}$. *Choose* $\bar{\rho}\in(0,0.5), \bar{\sigma} \in(\bar{\rho},1), \delta\in(0,1)$.
Step 1.: *If* $\Vert H(\mu^{k},w^{k}) \Vert ^{2}=0$, *then stop*.
Step 2.: *Find* $(\Delta\mu^{k},\Delta w^{k})$ *by solving* (19). *If* (28) *is not satisfied*, *then choose* $\tau_{k}$ *and compute*
  32 $$ \bigl(\Delta\mu^{k}, \Delta w^{k} \bigr)=- \bigl(\nabla H \bigl(\mu^{k},w^{k} \bigr)^{\top}{ \nabla H \bigl(\mu^{k},w^{k} \bigr)}+\tau_{k}I \bigr)^{-1}\nabla h \bigl(\mu^{k},w^{k} \bigr), $$
  *such that* (28) *is satisfied*.
Step 3.: *Find a step*-*length* $t^{k}\in(0,1]$ *satisfying* (29)-(31).
$\mu^{k+1}=\mu^{k}+t^{k}\Delta\mu^{k}, w^{k+1}=w^{k}+t^{k}\Delta w^{k}$. *Set* $k:=k+1$ *and go to Step* 1.

### Remark 2

In Step 2, if (28) is not satisfied, then the technique in [18], pp.264-265, is used to choose $\tau_{k}$. From Lemma 3.1 in [18], it is not difficult to find $t^{k}$ that can satisfy (29)-(31).

In order to obtain the global convergence of Algorithm 4.1, throughout the rest of this paper, we define the level set $\mathcal{L}(\mu^{0},w^{0})=\{(\mu,w)|h(\mu,w)\leq h(\mu ^{0},w^{0})\}$ for $(\mu^{0},w^{0})\in{\mathcal {R}}_{++}\times{\mathcal {R}}^{n+2m}$.

### Theorem 4.1

*Suppose that* $\nabla H(\mu,w)$ *is Lipschitz continuous in* $\mathcal {L}(\mu^{0},w^{0})$. *Then we have*

$$ \lim_{k\rightarrow\infty}\nabla h \bigl(\mu^{k}, w^{k} \bigr)=0. $$

### Proof

The proof follows Theorem 3.2 in [18] and condition (28). □

### Theorem 4.2

*Let* $(\mu^{0},w^{0})\in{\mathcal {R}}_{++}\times{\mathcal {R}}^{n+2m}$, $H(\mu,x,\lambda,z)$ *be defined by* (7). *Assume that* $\nabla H(\mu,w)$ *is Lipschitz continuous in* $\mathcal {L}(\mu^{0},w^{0})$, $t^{k}=1$ *is admissible and* (28) *is satisfied for all* $k\geq k_{0}$, $\nabla H(\mu^{*},w^{*})$ *is nonsingular where* $k_{0}$ *is sufficiently great*, *and* $(\mu^{*},w^{*})$ *is a limited point of* $\{(\mu^{k},w^{k})\}$ *generated by Algorithm* 4.1. *Then the sequence* $\{(\mu^{k},w^{k})\}$ *converges to* $(\mu^{*},w^{*})$ *quadratically*.

### Proof

From Theorem 4.1, we have

$$ \lim_{k\rightarrow\infty}\nabla h \bigl(\mu^{k}, w^{k} \bigr)=0, $$

where $\nabla h(\mu^{k}, w^{k})=\nabla H(\mu^{k}, w^{k})H(\mu^{k}, w^{k})$. That is, the sequence $\{(\mu^{k},w^{k})\}$ is convergent. Since $\nabla H(\mu^{*},w^{*})$ is nonsingular and $(\mu^{*},w^{*})$ is a limited point of $\{(\mu^{k},w^{k})\}$ generated by Algorithm 4.1, we have

$$ \lim_{k\rightarrow\infty}H \bigl(\mu^{k}, w^{k} \bigr)=0. $$

According to the assumption that there exists $k_{0}$ such that $t^{k}=1$ is admissible and (28) is satisfied for all $k\geq k_{0}$, $\{(\mu^{k},w^{k})\}$ can be generated by Algorithm 3.1 for $k> k_{0}$. We can get the conclusion from Theorem 3.1 directly. This completes the proof. □

## Numerical results

In this section, we present some numerical results for Algorithm 4.1. All codes are written in Matlab and run on a RTM i5-3210M personal computer. In the algorithm, we choose $\gamma=0.001$. We also use $\Vert H(\mu^{k},w^{k}) \Vert \leq10^{-5}$ as the stopping rule for all examples.

It is not easy to find proper test examples for the variational inequalities with nonlinear constraints. Hence, we modify some test examples in references and solve them by Algorithm 4.1.

### Example 5.1

see [19]

Let

$$ F(x)= \begin{pmatrix}2x_{1}+0.2x_{1}^{3}-0.5x_{2}+0.1x_{3}-4\\ -0.5x_{1}+x_{2}+0.1x_{2}^{3}+0.5\\ 0.5x_{1}-0.2x_{2}+2x_{3}-0.5 \end{pmatrix} $$

and

$$ g(x)=-x_{1}^{2}-0.4x_{2}^{2}-0.6x_{3}^{2}+1. $$

It is verified that the problem has the solution $x^{*}=(1, 0, 0)$ easily. The initial point is $x^{0}=(0,1,1)$ and $\mu^{0}=0.2$.

### Example 5.2

This example is derived from [20]. Because the original problem is an optimization problem, we give its form of variational inequalities by the optimality condition, i.e.,

$$ F(x)= \begin{pmatrix}2x_{1}-5\\ 2x_{2}-5\\ 4x_{3}-21\\ 2x_{4}+7 \end{pmatrix} ,\qquad g(x)=\begin{pmatrix}-x_{1}^{2}-x_{2}^{2}-x_{3}^{2}-x_{4}^{2}-x_{1}+x_{2}-x_{3}+x_{4}+8\\ -x_{1}^{2}-2x_{2}^{2}-x_{3}^{2}-2x_{4}^{2}+x_{1}+x_{4}+10\\ -2x_{1}^{2}-x_{2}^{2}-x_{3}^{2}-2x_{1}+x_{2}+x_{4}+5 \end{pmatrix}. $$

The solution of Example 5.2 is $x^{*}=(0,1,2,-1)$. The initial point is $x^{0}=(0,0,0,0)$ and $\mu^{0}=0.2$.

In Tables 1-2, ‘*k*’ means the number of iterations, ‘$\Vert H(\mu ^{k},w^{k}) \Vert $’ means the 2-norm of $H(\mu^{k},w^{k})$. From Tables 1-2, we can observe that Algorithm 4.1 can find the solution in a smaller number of iterations for the above two examples. In order to further show the efficiency of Algorithm 4.1, we give other two examples where the dimension of the problems is from 100 to 1,000.

**Table 1:** **Numerical results for Example** **5.1** **with** $\pmb{x^{0}=(0,1,1)}$

	**Example ** **5.1**			
***k***	$\boldsymbol {x_{1}} $	$\boldsymbol {x_{2}} $	$\boldsymbol {x_{3}} $	$\boldsymbol {\Vert H(\mu^{k},w^{k}) \Vert } $
1	1.6663	0.1527	0.7730	4.0941
2	1.6460	0.3416	0.0000	2.9087
3	1.6431	0.3212	0.0000	1.7601
4	1.2659	0.3957	0.0000	0.8018
5	1.0386	0.1050	0.0007	0.2139
6	1.0019	0.0174	0.0008	0.0301
7	1.0021	0.0005	0.0000	0.0043
8	1.0003	0.0000	0.0000	7.2097e–04
9	1.0000	0	0	1.8416e–05
10	1.0000	0.0000	0.0000	9.6147e–06

**Table 2:** **Numerical results for Example** **5.2** **with** $\pmb{x^{0}=(0,0,0,0)}$

	**Example ** **5.2**				
***k***	$\boldsymbol {x_{1}} $	$\boldsymbol {x_{2}} $	$\boldsymbol {x_{3}} $	$\boldsymbol {x_{4}} $	$\boldsymbol {\Vert H(\mu^{k},w^{k}) \Vert } $
1	0.3330	1.0661	4.0792	−4.3984	0.7137
2	3.7727	3.2371	5.7749	−2.3704	0.4308
3	1.8029	2.4677	2.9939	−1.3529	0.1151
4	0.6067	1.7571	2.2497	−0.9003	0.0399
5	0.4095	1.1936	2.1080	−0.7000	0.0076
6	0.0093	1.0500	2.1182	−0.8442	0.0045
7	0.0009	0.9994	2.0014	−0.9991	7.8889e–04
8	0.0002	1.0000	2.0005	−1.0000	3.2619e–05
9	0.0000	1.0000	2.0000	−1.0000	1.3066e–06

In the following tests, we solve Δ*w* of the linear systems by using GMRES(m) package with $m=10$, allowing a maximum of 100 cycles (2,000 iterations). And we choose $\mu^{0}$ as a random number in $(0,5)$.

### Example 5.3

We consider the problem with nonlinear constraints. The problem is derived from [21] with different sizes. Based on the linear constraints of the original problem, we also add some nonlinear constraints to the problem. In this example,

$$\begin{aligned} F(x)= \begin{pmatrix}4& -2& \cdots& 0 &0 \\ 1 &4 &\cdots& 0 & 0\\ &&\cdots&&\\ 0 &0 &\cdots& 4&-2\\ 0 &0 &\cdots& 1 & 4 \end{pmatrix} \begin{pmatrix} x_{1}\\x_{2}\\x_{3}\\ \vdots\\ x_{n} \end{pmatrix} + \begin{pmatrix} -1\\-1\\-1\\ \vdots\\ -1 \end{pmatrix},\qquad g(x)=\begin{pmatrix} x_{1}+10\\ x_{2}+10\\ \cdots\\ x_{n}+10\\ 100-x_{1}^{2}\\ 100-x_{2}^{2}\\ \cdots\\ 100-x_{n}^{2} \end{pmatrix} . \end{aligned}$$

### Example 5.4

The example is the NCP. $F(x)=D(x)+Mx+q$. The components of $D(x)$ are $D_{j}(x)=d_{j}\cdot \operatorname{arctan}(x_{j})$, where $d_{j}$ is a random variable in $(0,5)$. The matrix $M=A^{\top}A+B$, where *A* is an $n\times n$ matrix whose entries are randomly generated in the interval $(-5,5)$, and the skew-symmetric matrix *B* is generated in the same way. The vector *q* is generated from a uniform distribution in the interval $(-50,50)$.

In Tables 3-4, ‘*n*’ means the dimension of problems, ‘No.it’ means the number of iterations, ‘CPU’ means the cpu time in seconds. ‘$\Vert H(\mu^{k},w^{k}) \Vert $’ means the 2-norm of $H(\mu^{k},w^{k})$. From Tables 3-4, we find that Algorithm 4.1 is robust to the different sizes for these two problems. Moreover, the iterative number is insensitive to the size of problems in our algorithm. In other words, our algorithm is more effective for two problems.

**Table 3:** **Numerical results for Example** **5.3**

	**Example ** **5.3**		
***n***	**No.it**	**CPU**	$\boldsymbol {\Vert H(\mu^{k},w^{k}) \Vert } $
100	8	0.3120	7.7321e–06
200	8	0.4992	2.9356e–06
300	9	0.7956	2.5000e–06
400	9	1.1700	1.3077e–06
600	9	2.1840	1.8843e–06
800	9	3.8220	2.1879e–06
1,000	9	5.6004	2.0789e–06

**Table 4:** **Numerical results for Example** **5.4**

	**Example ** **5.4**		
***n***	**No.it**	**CPU**	$\boldsymbol {\Vert H(\mu^{k},w^{k}) \Vert } $
100	8	0.4992	3.5558e–07
200	8	1.1388	3.0105e–06
300	8	2.3244	4.7536e–06
400	8	4.5552	7.7267e–06
600	9	13.1665	8.8521e–07
800	9	25.3346	1.4537e–06
1,000	9	41.4027	2.0736e–06

## Conclusions

Based on the framework of smoothing Newton method, we propose a new smoothing inexact Newton algorithm for variational inequalities with nonlinear constraints. Under some mild conditions, we establish the global and local quadratic convergence. Furthermore, we also present some preliminary numerical results which show efficiency of the algorithm.
